# Physiological clearance of Aβ by spleen and splenectomy aggravates Alzheimer‐type pathogenesis

**DOI:** 10.1111/acel.13533

**Published:** 2021-12-23

**Authors:** Zhong‐Yuan Yu, Dong‐Wan Chen, Cheng‐Rong Tan, Gui‐Hua Zeng, Chen‐Yang He, Jun Wang, Xian‐Le Bu, Yan‐Jiang Wang

**Affiliations:** ^1^ Department of Neurology and Centre for Clinical Neuroscience Daping Hospital Third Military Medical University Chongqing China; ^2^ Institute of Brain and Intelligence Third Military Medical University Chongqing China; ^3^ Chongqing Key Laboratory of Ageing and Brain Diseases Chongqing China

**Keywords:** Alzheimer's disease, Aβ burden, behaviour deficits, splenectomy

## Abstract

**Background:**

A previous study demonstrated that nearly 40%–60% of brain Aβ flows out into the peripheral system for clearance. However, where and how circulating Aβ is cleared in the periphery remains unclear. The spleen acts as a blood filter and an immune organ. The aim of the present study was to investigate the role of the spleen in the clearance of Aβ in the periphery.

**Methods:**

We investigated the physiological clearance of Aβ by the spleen and established a mouse model of AD and spleen excision by removing the spleens of APP/PS1 mice to investigate the effect of splenectomy on AD mice.

**Results:**

We found that Aβ levels in the splenic artery were higher than those in the splenic vein, suggesting that circulating Aβ is cleared when blood flows through the spleen. Next, we found that splenic monocytes/macrophages could take up Aβ directly in vivo and in vitro. Splenectomy aggravated behaviour deficits, brain Aβ burden and AD‐related pathologies in AD mice.

**Conclusion:**

Our study reveals for the first time that the spleen exerts a physiological function of clearing circulating Aβ in the periphery. Our study also suggests that splenectomy, which is a routine treatment for splenic rupture and hypersplenism, might accelerate the development of AD.

## Graphical abstract

Our study reveals the physiological capacity of the spleen to clear circulating Aβ, and the splenectomy aggravates Alzheimer‐type pathogenesis in APP/PS1 transgenic mice.

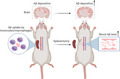

**Abbreviations**
Aß: amyloid‐ß
AD: Alzheimer’s disease
APC: allophycocyanin
APP: amyloid precursor protein
CAA: cerebral amyloid angiopathy
FA: formic acid
FBS: foetal bovine serum
GFAP: glial fibrillary acidic protein
Iba1: Ionized calcium binding adaptor molecule 1
Map‐2: microtubule‐associated protein‐2
MWM: Morris water maze
NC: nitrocellulose
NOR: Novel Object Recognition
SDS: sodium dodecyl sulfonate
TBS: Tris buffer solution

## 1 INTRODUCTION

Alzheimer's disease (AD) is the most common type of dementia characterized by progressive memory loss and cognitive deficits. Currently, there are no available disease‐modifying treatments to halt or slow the progression of AD (Cheng, et al., 2020). Extracellular senile plaques composed of amyloid‐β (Aβ) are a main hallmark of AD. Aβ overproduction and impaired Aβ clearance have been suggested to play causative or pivotal roles in AD pathogenesis (Hardy & Selkoe, 2002; Selkoe & Hardy, 2016). However, only 1% of all AD cases are familial AD cases caused by overproduction of Aβ due to mutations in the amyloid precursor protein (APP) and PS1/2 genes. Dysfunction of Aβ clearance is believed to be the main cause of Aβ accumulation in sporadic AD, which accounts for 99% of all AD cases (Selkoe & Hardy, 2016). Thus, more attention should be paid to targeting Aβ clearance for the treatment of AD.

It is traditionally believed that Aβ is mainly cleared in the brain, such as through phagocytosis by microglia and subsequent autophagic elimination (Boland et al., 2018). However, convincing evidence has indicated that the peripheral system plays a role in the clearance of Aβ (Cheng & Wang, 2020; Jin et al., 2017; Wang et al., 2017; Xiang et al., 2015). Our and others' studies suggest that approximately 40%–60% of brain‐derived Aβ is transported to the peripheral system for clearance (Xiang et al., 2015; Yuede et al., 2016b). However, the specific peripheral organs and tissues involved in Aβ clearance are currently unclear.

Similar in the structure to a large lymph node, the spleen acts primarily as a blood filter and exerts immunological functions. It removes old red blood cells and holds a reserve of blood. The spleen is composed of a variety of immune cells, with 7%–8% of all cells being monocytes/macrophages. Monocytes/macrophages are an important part of the innate immune system and function as the first line of defence in the host through various effector functions (Swirski et al., 2009). The capacity of blood monocytes/macrophages to clear Aβ has been confirmed (Gu et al., 2016). Moreover, their ability to clear circulating Aβ is significantly decreased in patients with AD and during the course of ageing (Chen et al., 2020). Recent study found spleen is involved in the senescence and ageing of solid organs indicated by the transplantation of splenocytes from aged mice into young mice (Yousefzadeh et al., 2021). Whether the spleen has the physiological capacity to clear Aβ, and its pathophysiological relevance to AD remains largely unknown.

In the present study, we investigated the physiological clearance of Aβ by the spleen and established a mouse model of AD and spleen excision by removing the spleens of APP/PS1 mice. We found that splenic monocytes/macrophages could take up Aβ directly in vivo and in vitro and that the removal of the spleen increased the brain Aβ burden in AD mice. In addition, we found that spleen excision decreased the number of monocytes/macrophages and resulted in higher levels of Aβ in the blood.

## 2 RESULTS

### 2.1 Physiological clearance of circulating Aβ by the spleen

**FIGURE 1:**
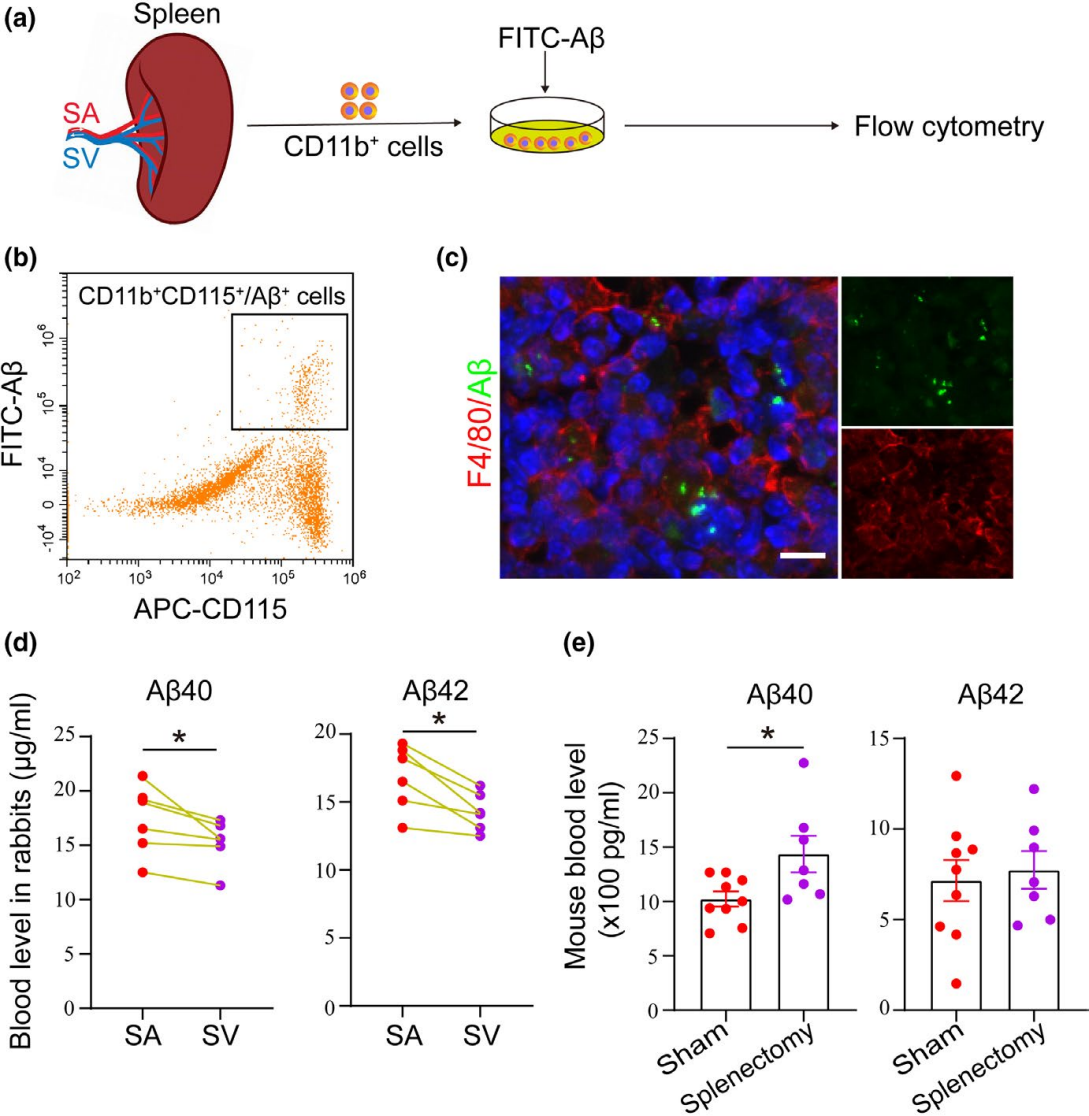
Physiological capacity for the spleen to clear Aβ. (a and b) Schematic diagram and analysis of Aβ clearance by splenic monocytes/macrophages in vitro. (c) Representative images of Aβ uptake by monocytes/macrophages in the mouse spleen after injection of FITC‐Aβ into the tail vein. (d) Comparison of Aβ levels in the splenic arteries (SAs) and splenic veins (SVs) of rabbits. E. Aβ levels in the blood of mice that underwent splenectomy and sham control mice. n = 6–9 per group; *Indicates *p* < 0.05. Scale bar 100 µm. The error bars are the SEMs

Firstly, to explore whether the splenic monocytes could uptake the Aβ, we isolated the splenic monocytes/macrophages by CD11b‐conjugated magnetic beads and cultured with FITC‐labelled Aβ for an hour. We found that splenic monocytes/macrophages phagocytosed FITC‐conjugated Aβ in vitro and directly took up Aβ after FITC‐conjugated Aβ was injected into the tail vein (Figure 1a‐c). Then, we collected the blood from rabbit splenic artery and vein, and compared the circulating Aβ levels in the rabbit splenic artery and vein. Interestingly, the blood levels of Aβ42 and Aβ40 in the rabbit splenic vein were 11.7% and 15.2% lower, respectively, than those in the rabbit splenic artery (t = 2.64, *p *= 0.046 for Aβ40; t = 2.26, *p *= 0.47 for Aβ42; Figure 1d). To investigate the roles of spleen in the clearance of Aβ from blood, we conducted splenectomy of APP/PS1 mouse at the age of 4 months and detected the blood levels of Aβ40 and Aβ42 at 9 months of age. We found that blood Aβ40 levels were statistically higher in AD mice underwent splenectomy than that in sham AD mice, and blood Aβ42 levels were also higher in splenectomy AD mice than sham mice but the increase did not reach statistical significance (t = 2.48, *p *= 0.027 for Aβ40; t = 0.36, *p *= 0.72 for Aβ42; Figure 1e). Taken together, our data suggested that monocytes/macrophages in the spleen could physiologically clear circulating Aβ.

### 2.2 Spleen excision impaired behaviour performance in AD mice

**FIGURE 2:**
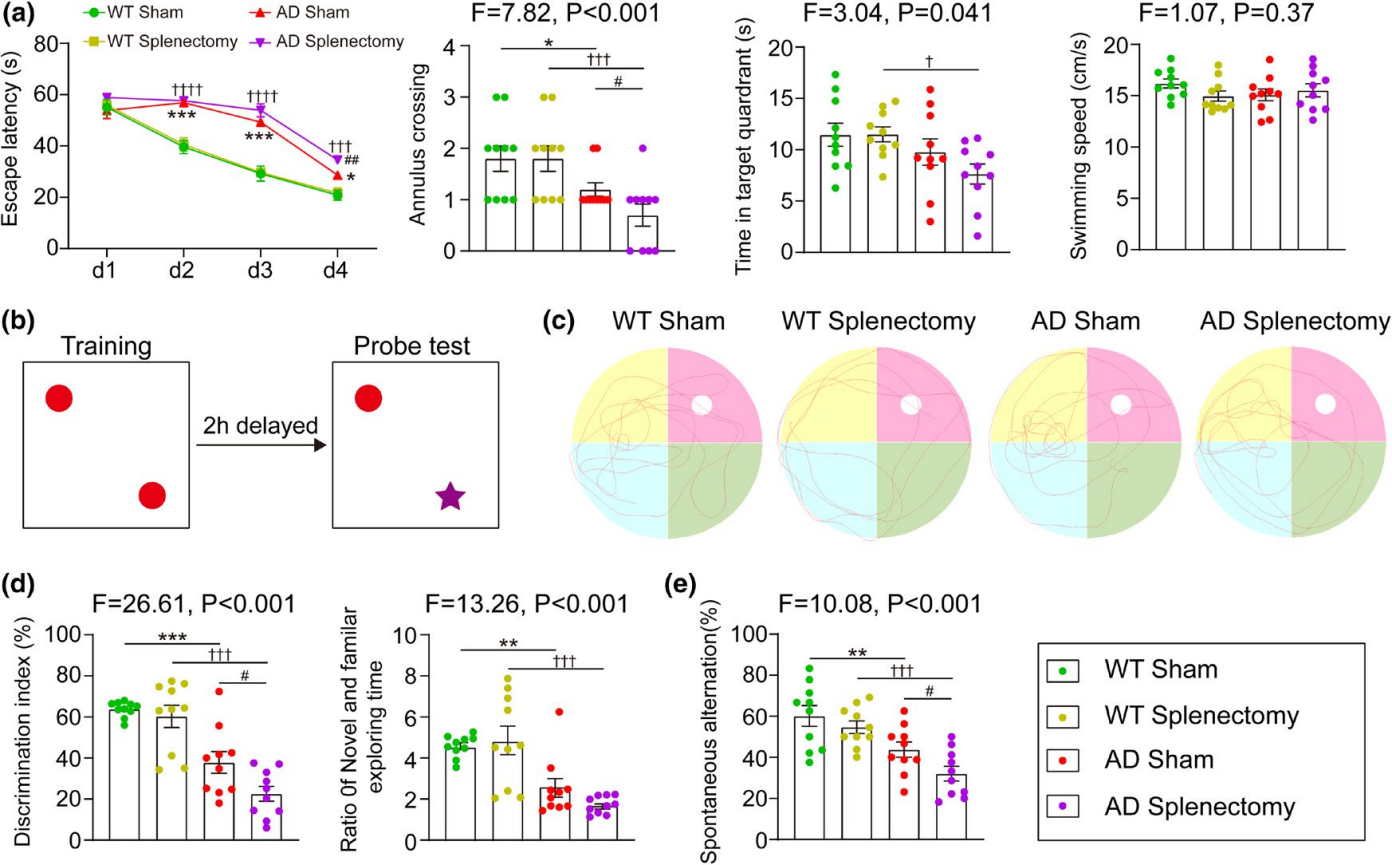
Effect of spleen excision on behaviour performance in AD mice. (a) The escape latency, annulus crossing, time in target quadrant and swimming speed in the Morris water maze (MWM) test. (b, d) The schematic image, statistics of discrimination index and ratio of time novel and time familiar in novel object test. (c) The representative images of MWM test in each group. (e) Spontaneous alterations in the Y maze test. n = 10 per group. Compared with WT Sham group, *indicates *p* < 0.05, **indicates *p* < 0.01, ***indicates *p* < 0.001; Compared with WT Splenectomy group, ^†^indicates *p* < 0.05, ^†††^indicates *p* < 0.001, ^††††^indicates *p* < 0.0001; Compared with AD Sham group, ^#^indicates *p* < 0.05, ^##^indicates *p* < 0.01. The error bars are the SEMs

To evaluate the change of learning ability and memory after splenectomy, the Morris water maze (MWM) test, Y maze test and Novel Object Recognition (NOR) test were conducted 5 months after splenectomy. In MWM test, there is no difference in escape latency, annulus crossing, time in target quadrant and swimming speed in probe test between WT sham group and WT splenectomy group, indicating the splenectomy itself did not affect the spatial learning ability (Figure 2a, c). However, AD mice received splenectomy presented impaired spatial learning ability, as reflected by a significant increase in the escape latency at day 4 (*p *= 0.0078, Figure 2a, c), and worsen memory consolidation, as reflected by fewer number of platform area crossings compared with AD sham group (*p *= 0.046, Figure 2a, c). We also conducted the NOR test to the investigate short‐term recognition memory. There was no difference in the preference for new objects between WT sham and WT splenectomy groups (*p *= 0.36, Figure 2b, d). However, the discrimination index of AD mice was reduced compared with WT mice, while splenectomy significantly worsens the reduction in AD mice (AD sham vs. AD splenectomy, *p *= 0.019; Figure 2b, d). In Y maze test, compared with AD sham mice, mice that underwent splenectomy displayed worse memory, as indicated by the decreased percentage of spontaneous alterations (AD sham vs. AD splenectomy, *p *= 0.041; Figure 2e). Together, these findings showed that splenectomy significantly worsens memory deficits in AD mice.

### 2.3 Changes in the proportions of peripheral blood immune cells in AD mice after spleen excision

**FIGURE 3:**
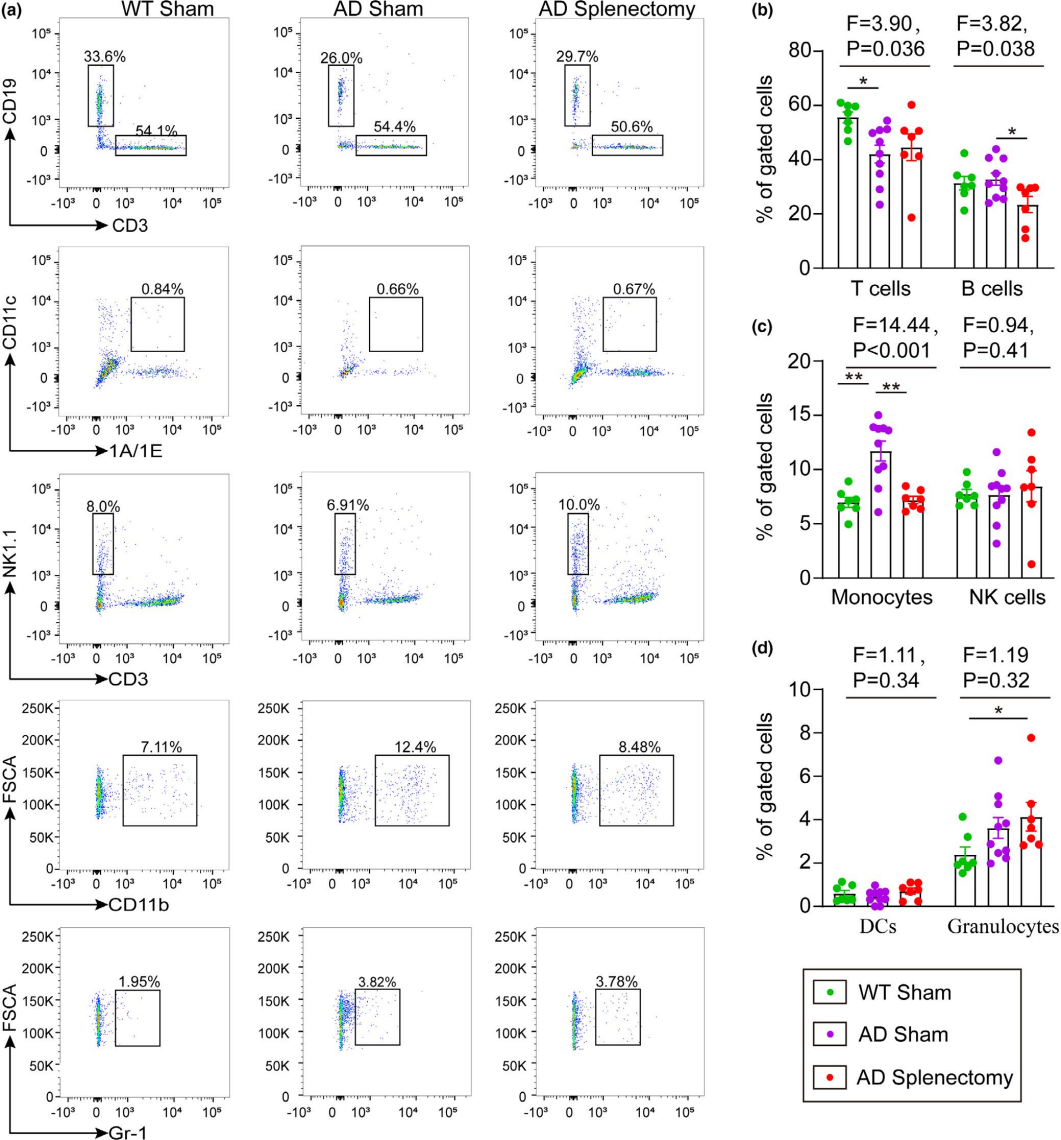
Effect of spleen excision on the proportions of peripheral blood immune cells in AD mice. (a) Representative images of flow cytometry analysis of blood immune cells. (b) Proportions of T cells and B cells in the peripheral blood of wild type, AD sham mice and AD mice that underwent splenectomy. (c) Proportions of monocytes and NK cells in peripheral blood of wild type, AD sham mice and AD mice that underwent splenectomy. (d) Proportions of DCs and granulocytes in peripheral blood of wild type, AD sham mice and AD mice that underwent splenectomy. DCs denotes dendrite cells, WT denotes wild type. n = 7–9 per group; *indicates *p* < 0.05. The error bars are the SEMs

We investigated the proportions of peripheral blood immune cells in AD mice after splenectomy. Our data showed that the proportion of T cells in the AD sham control group was lower than that in WT mice (AD sham vs. WT, *p *= 0.13; Figure 3), but there were no differences between the AD sham control and AD splenectomy groups (*p *= 0.629, Figure 3). Additionally, the proportion of monocytes was higher in AD sham control mice than in WT mice and lower in AD splenectomy mice than WT mice (AD sham vs. WT, *p *= 0.002; AD sham vs. AD splenectomy, *p *= 0.002; Figure 3). Compared with those in WT mice, the proportions of other cells, including B cells, NK cells, DC cells and granulocytes, in the AD sham group and AD splenectomy group were not different (*p *> 0.05, Figure 3).

### 2.4 Spleen excision aggravates the Aβ burden in the brains of AD mice

**FIGURE 4:**
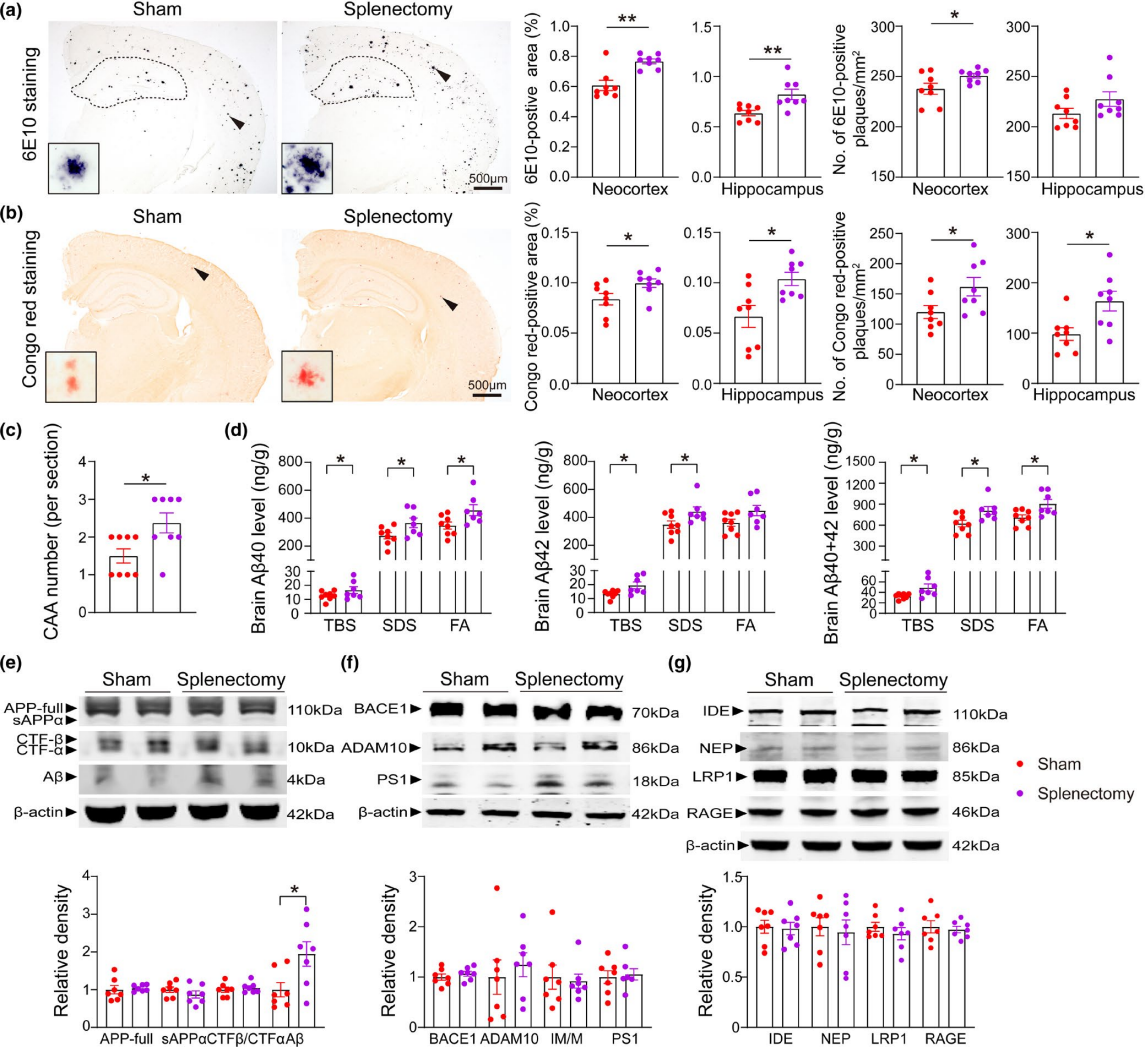
Spleen excision aggravates Aβ burden in AD mice. (a and b) Immunostaining and quantification of Aβ plaques stained with 6E10 and Congo red in the neocortices and hippocampi of AD mice with or without splenectomy. (c) Statistical analysis of cerebral amyloid angiopathy (CAA) number, as determined by Congo red staining. (d) Comparison of Aβ40 and Aβ42 levels in the TBS, SDS and FA fractions of brain homogenates between the sham group and splenectomy group. (e) Representative Western blots and quantitative analysis of the levels of APP and its metabolites (sAPPα, CTFβ, CTFα, and Aβ) in brain homogenates. (f) Representative Western blots and quantitative analysis of the levels of APP‐metabolizing enzymes in brain homogenates. (g) Representative Western blot and quantitative analysis of the levels of Aβ‐degrading enzymes and Aβ‐transporting receptors in brain homogenates. n = 6–9 per group; *indicates *p* < 0.05; **indicates *p* < 0.01. The scale bars in a and b are 500 µm. The error bars are the SEMs

We performed Aβ 6E10 staining to detect total Aβ plaques and Congo red staining to detect compact Aβ plaques. Compared with sham control mice, AD mice underwent splenectomy displayed a larger percentage area of both 6E10‐positive in the neocortex and hippocampus (U = 8, *p *= 0.0096 for the neocortex; t = 3.22, *p *= 0.0062 for the hippocampus; Figure 4a, b). AD mice underwent splenectomy showed an increase of brain compact Aβ plaques stained by Congo red compared to AD sham group (t = 2.25, *p *= 0.041 for the neocortex; t = 2.93, *p *= 0.011 for the hippocampus; Figure 4a, b). We also examined the change of amyloid deposition in brain blood vessels after splenectomy in AD mice. We found that AD mice underwent splenectomy exhibited more Aβ deposition on vessel walls in the brain, which indicates the development of cerebral amyloid angiopathy (CAA), than AD sham control mice (U = 12, *p *= 0.046; Figure 4c).

To match the immunohistochemical results, we conducted the ELISAs to detect the different forms of Aβ in brain. Consistently, AD mice underwent splenectomy had higher levels of total Aβ, Aβ42 and Aβ40 in the TBS, SDS and FA fractions of brain homogenates than AD sham controls (*p *< 0.05 for all data, Figure 4d). To reveal the potential mechanism underlying the increased Aβ burden in the brain, we investigated Aβ production and the levels of Aβ transport‐related proteins. There were no differences in the levels of APP itself or its metabolites, including CTFβ, CTFα, sAPPα, BACE1, ADAM10 and PS1, between AD mice underwent splenectomy and sham control mice (*p *> 0.05 for all data, Figure 4e), but the Aβ monomer level in AD mice underwent splenectomy was higher than that in sham control mice (t = 2.36, *p *= 0.036; Figure 4e, f). In addition, there were no differences in the levels of Aβ transport‐ and degradation‐related proteins, including IDE, NEP, LRP1 and RAGE, between AD mice underwent splenectomy and AD sham control mice (*p *> 0.05 for all data, Figure 4g). Taken together, these data suggest that the increased Aβ burden was not attributed to changes in Aβ production and clearance in the brain.

### 2.5 Spleen excision aggravates AD‐type pathologies in AD mice

**FIGURE 5:**
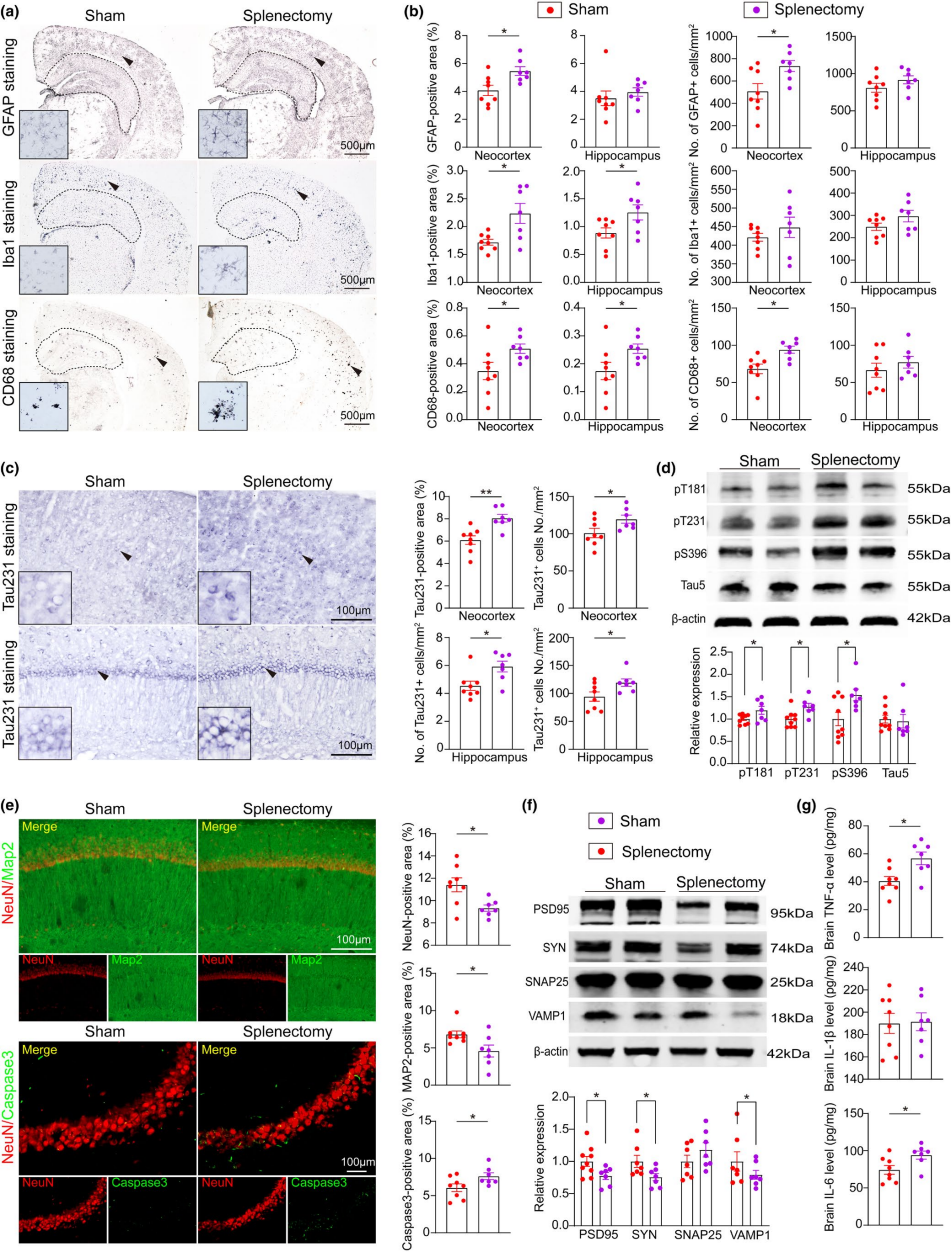
Spleen excision aggravates AD‐related pathogenesis. (a and b) Immunostaining of astrocytes and microglia with GFAP, Iba1 and CD68 in the neocortices and hippocampi of AD mice with or without splenectomy and quantification. (c) Representative immunostaining images and quantification of pT231 expression. (d) Representative Western blots and quantitative analysis of phosphorylated Tau protein (pT181, pT231 and PS396) and total Tau protein levels in brain homogenates. (e) Representative images and quantification of neurons (NeuN, red) and dendrites (MAP2, green) in the CA1 region of the hippocampus and neural apoptosis (Caspase3, green) in the CA3 region of the hippocampus. (f) Representative images and quantification of synaptic proteins (PSD95, Synapsin‐1, SNAP25 and VAMP1). (g) Statistical analysis of the levels of inflammatory factors, including TNF‐α, IL‐1β and IL‐6. n = 6–9 per group; *indicates *p* < 0.05; **indicates *p* < 0.01. The scale bars in a and b are 500 µm, and the scale bar in e is 100 µm. The error bars are the SEMs

To explore the inflammatory response after spleen excision, we conducted the GFAP, Iba1 and CD68 staining to label astrocytes, total microglia and activated microglia, respectively. Compared with sham control mice, AD mice underwent splenectomy exhibited more astrocytes in the neocortex, more total microglia in both the neocortex and hippocampus, and more activated microglia in the neocortex (*p *< 0.05 for all data, Figure 5a, b), and increased levels of proinflammatory factors, including TNF‐α and IL‐6 (t = 2.98, *p *= 0.011 for TNF‐α; t = 2.46, *p *= 0.029 for IL‐6; Figure 5g).

Tau phosphorylation is also a hallmark of AD, we further explored the change of tau phosphorylation after splenectomy. There were more phospho‐tau (pT231)‐positive neurons in both the neocortex and hippocampus in AD mice underwent splenectomy than in sham mice (t = 3.74, *p *= 0.0025 for the neocortex; t = 2.73, *p *= 0.017 for the hippocampus; Figure 5c). The extent of tau phosphorylation at multiple epitopes, including Thr181 (pT181), Thr231 (pT231) and pS396, was increased in AD mice underwent splenectomy compared with sham mice, whereas no difference was observed in the levels of total tau (defined as tau5) between the two groups (t = 2.24, *p *= 0.042 for pT181; t = 2.88, *p *= 0.012 for pT231; t = 2.64, *p *= 0.020 for PS396; t = 0.29, *p *= 0.77 for Tau5; Figure 5d).

Compared with sham mice, AD mice underwent splenectomy displayed less staining for markers of viable neurons (NeuN) and dendrites (Map2) (t = 2.89, *p *= 0.013 for NeuN; U = 9, *p *= 0.029 for Map2; Figure 5e) and more staining for an apoptosis‐related protein (activated caspase‐3) in the hippocampus (t = 2.21, *p *= 0.045; Figure 5e). The levels of synapse‐related proteins, including PSD95, SYN and VAMP1, but not SNAP25, were reduced in the brains of AD mice underwent splenectomy compared to those in sham mice (t = 2.28, *p *= 0.039 for PSD95; t = 2.23, *p *= 0.046 for SYN; U = 16, *p *= 0.032 for VAMP1; t = 1.21, *p *= 0.25 for SNAP25; Figure 5f).

Taken together, our findings suggest that splenectomy worsens neuroinflammation, tau hyperphosphorylation and neurodegeneration in the brain of AD mice.

## 3 DISCUSSION

Previous studies suggested that the peripheral system physiologically clears circulating Aβ derived from the brain (Qosa et al., 2014; Xiang et al., 2015; Yuede et al., 2016a). However, the catabolism of circulating Aβ in the periphery remains largely unclear (Cheng et al., 2020; Wang et al., 2017). It has been shown that there is Aβ deposition in the spleen (Di Benedetto et al., 2019), implying that spleen might be involved in Aβ metabolism in the periphery. In the present study, Aβ level in the blood of splenic artery was higher than that in the splenic vein in rabbit whose Aβ amino‐acid sequence is identical to that of humans, indicating that Aβ was cleared when it circulates through the spleen. Consistently, endogenous Aβ levels in the blood were increased after splenectomy in old wild‐type mice (Kong et al., 2017). These findings suggest that spleen physiologically clears Aβ from the blood.

As a blood filter and immune organ, spleen is composed of a variety of immune cells, with monocytes/macrophages accounting for 7%–8% of total cells. Monocytes and macrophages have been suggested to be able to clear circulating Aβ (Chen et al., 2020; Gu et al., 2016). In the present study, we found that macrophages/monocytes could take up Aβ in vitro and in vivo, suggesting that monocyte/macrophage in the spleen also function to clear circulating Aβ. A main function of innate immune is to clear pathological substances from the body. Large amount of evidence suggests that microglia is a main scavenger of brain Aβ, and its dysfunction is involved in the pathogenesis of AD (Condello et al., 2015; Keren‐Shaul et al., 2017). However, the roles of monocytes and macrophages, the counters part of microglia in the periphery, in the pathogenesis of AD remain largely unknown. A recent genome‐wide meta‐analysis found that AD risk‐associated genes are strongly expressed in immune‐related tissues and cell types including spleen, liver and microglia (Jansen et al., 2019). These findings imply that monocytes and macrophages in the spleen would be also involved in AD pathogenesis.

To explore the roles of spleen in the clearance of Aβ from blood and brain, we removed the spleens of AD mice at 4 months of age, when no obvious Aβ plaques were present in the brain of APP/PS1 mice, and investigated brain Aβ burden 5 months after spleen excision. We found that blood Aβ40 levels were statistically higher in AD mice underwent splenectomy than that in sham AD mice, indicating that peripheral clearance of Aβ40 decreased after spleen excision. In our study, blood Aβ42 levels were also higher in splenectomy AD mice than sham mice but the increase did not reach statistical significance. Aβ42 level in the blood is lower than that of Aβ40, as Aβ42 is more prone to aggregate in the brain than Aβ40. As a result, less amount of brain Aβ42 effluxes to blood in relative to Aβ40. This would lead to the less amount of Aβ42 taken up by spleen cells than Aβ40. This may explain why the extent of increase in blood Aβ42 was less than that of Aβ40 after splenectomy. However, the chronic low levels of Aβ42 clearance in blood could be also meaningful, as it is estimated that a subtle 2–5% reduction of Aβ clearance efficiency would be enough to lead to brain Aβ accumulation and the development of AD (Roberts et al., 2017).

In the present study, total brain Aβ burden was increased by 13.1% as measured by ELISA after spleen excision, implying that the spleen plays a substantial role in clearing brain‐derived Aβ. The increase of Aβ burden in the brain after splenectomy may be due to two main mechanisms. One is that removal of spleen decreased the clearance of circulating Aβ by spleen. Another mechanism could be related to the changes of blood immune cells after splenectomy. Previous studies have showed splenectomy changed the number of blood immune cells in human and animals, with a decrease of T cells, B cells and monocytes/macrophages (Chen et al., 2021; Rosado et al., 2013; Tomczyk et al., 2017; Wasserstrom et al., 2008). Similarly, both monocytes and B cells in blood decreased in AD mice underwent splenectomy in relative to control AD mice in our study. Decrease in number of monocytes may reduce their uptake of circulating Aβ, and decrease in number of B cells would lead to less production of anti‐Aβ autoantibodies, which are suggested to be able to remove Aβ deposition from brain, such as aducanumab (Sevigny et al., 2016).

Based on our findings, more attention should be paid to the potential role of spleen in the development of AD. A recent study found that aged spleen cells accelerate the process of ageing, which is an important risk factor of AD (Yousefzadeh et al., 2021). Splenectomy is a routine treatment for splenic rupture and hypersplenism. It increases the risk of various immune‐related diseases, such as infections, tumours and type 2 diabetes (Camprubí et al., 2019; Casciani et al., 2020). Our study indicates that removal of spleen accelerates the development of AD in mice. Dysfunction of the immune system is involved in AD pathogenesis (Golde, 2016). To our knowledge, the impacts of spleen excision on cognition in humans have been rarely studied thus far. Therefore, studies are needed to investigate whether excision of the spleen increases the risk of AD development in subjects who received splenectomy.

It has been reported that the ability of monocytes to clear circulating Aβ is decreased along with ageing and further reduced in AD patients (Chen et al., 2020). A potential approach to prevent AD is to enhance the Aβ‐phagocytosis function of monocytes/macrophage. A recent study showed the enhancing of monocyte Aβ processing by polysaccharide krestin attenuates AD‐type pathology and cognitive deficits (Chen et al., 2021). In addition, transplantation with spleen cells from young mice could delay the course of ageing in old mice (Yousefzadeh et al., 2021). These findings suggest that spleen might be a potential therapeutic target for AD.

In conclusion, our study revealed the physiological capacity of the spleen to clear circulating Aβ. The pathophysiological impacts of spleen disorders on brain health and whether the spleen is a potential therapeutic target for AD need to be investigated in future.

## 4 EXPERIMENTAL PROCEDURES

### 4.1 Animals

Female APPswe/PS1dE9 transgenic mice on C57BL/6 background were obtained from Jackson Laboratory (Bar Harbor, ME, USA, JAX catalog number: 005864). They carry two transgenes with AD‐linked mutations: a chimeric mouse/human APP695 with the Swedish mutation (K595N/M596L) and human PS1 lacking exon 9 (PS1dE9), both are under the control of the prion promoter (Jankowsky et al., 2001; Ordonez‐Gutierrez et al., 2016). Female C57BL/6J (wild type, WT) mice were provided by the Animal Centre of Daping Hospital affiliated with the Third Military Medical University. All experimental procedures were approved by the Laboratory Animal Welfare and Ethics Committee of Daping Hospital. A total of 6 3‐month‐old female rabbits were used to investigate differences in Aβ levels between the splenic artery and splenic vein. The rabbits were anaesthetized with 5 ml/kg 25% urethane. Five millilitres of blood were collected from the splenic artery and from the splenic vein. All animals were randomly divided into different groups and housed in a temperature‐controlled room with a standard 12‐h light/ 12‐h dark cycle, and ad libitum access to food and water.

### 4.2 Splenectomy

Four‐month‐old AD mice and WT mice underwent splenectomy. In brief, the mice were anaesthetized by intraperitoneal injection of ketamine (100 mg/kg), and the skin was disinfected with iodophor three times. An approximately 2 cm long incision was made in the centre of the abdomen. After exposing the spleen, the upper pole of the spleen and blood vessels was gently ligated with sutures, and the spleen was removed. If there was no bleeding at the vascular ligation site, the abdominal cavity was sutured layer by layer. After disinfecting the skin with iodophor, the mice were placed in a warm place for thermal resuscitation. In the sham operation, the abdominal cavity was opened but splenectomy was not performed, and the abdominal cavity was directly sutured.

### 4.3 Behavioural tests

Five months after spleen excision, all experimental mice were subjected to the NOR test, Y maze and MWM test to investigate behaviour performance according to previous studies (Chen et al., 2021; Pakavathkumar et al., 2017). The NOR tests were conducted to analyse the short‐term memory. In training session, mice were placed in the box containing two identical objects in the diagonal two corners and allowed to explore for 5 min. After a 2h retention period, one object was replaced with a novel object in the original location, and the mice were resubjected to the box and allowed to explore for another 5 min. The time spent exploring and sniffing each object was recorded. The results are expressed as the discrimination index, which refers to [(Time novel − Time familiar)/(Time novel+Time familiar)×100%]. The Y maze consisted of three identical arms connected in the centre at a 120 degrees angle. The Y maze test was conducted to analyse the spontaneous alternations. Briefly, an experimental mouse was placed in a randomly selected arm and allowed to explore the apparatus freely. The sequence in which the mouse entered the three arms was recorded, and spontaneous alternations were analysed. For MWM test, the water maze was consisted of a circular pool (100 cm in diameter) and a platform (9cm in diameter) submerged 1.0cm under the warm water. In the training test, mice were subjected to the water maze to seek for the platform and were allowed to stay on the platform for 10s. The mice were guided to the platform if the mice were unable to locate the platform. All experiment mice were trained twice per day for four consecutive days. In the probe test, the platform was withdrawn and mice were resubjected to the water maze for exploring 60s. The number of crossing the platform, the path taken and the time spent in target quadrant was recorded and analysed. Performance was tracked with a computer tracking system (ANY‐maze; Stoelting).

### 4.4 Brain sampling

After the behavioural tests, all experimental mice were sacrificed, and brain and blood samples were collected according to our previous methods (Wang et al., 2018). Briefly, after anaesthetized, blood was drawn from the eyes and centrifuged at 1800xg for 10 min. After intracardial perfusion with 0.1% NaNO_2_ in normal saline, the right brain hemisphere was dissected and fixed with 4% paraformaldehyde, and coronal sections were cut with a frozen slicer at a thickness of 30 μm. The left brain hemisphere was snap‐frozen in liquid nitrogen, ground into a powder, divided into three vials, weighed and stored at −80°C for biochemical analysis.

### 4.5 Immunohistochemistry and Immunofluorescence

#### 4.5.1 In vivo Aβ uptake by splenic monocytes/macrophages

100 µl FITC‐Aβ (20 µg/ml) was injected into the tail vein of AD mice, and the spleen was sampled 1 hour after injecting. 10 µm spleen sections were stained with F4/80 antibody (1:200, Biolegend). Double immunofluorescence staining for FITC‐Aβ and F4/80 was defined as the positive uptake.

### 4.6 Measurement of Aβ plaques and cerebral amyloid angiopathy (CAA)

Five sections spanning the entire brain were stained with a 6E10 antibody (1:1000, Biolegend) or Congo red (Sigma) to detect Aβ plaques in the brain (the 6E10 antibody was used to stain compact and diffuse Aβ; Congo red was used to stain for compact Aβ). CAA was manually assessed in Congo red‐stained hippocampal sections under a microscope (Wilcock et al., 2006).

### 4.7 Investigation of Tau pathology, neuroinflammation and neurodegeneration

We conducted pT231 staining with an anti‐pT231‐tau antibody (1:400, Signalway antibody) to detect phosphorylated tau. Astrocytes were stained with an anti‐glial fibrillary acidic protein (GFAP) antibody (1:500, Abcam). Ionized calcium‐binding adaptor molecule 1 (Iba1) staining and CD68 staining were used to detect total and activated microglia, respectively (Iba1: 1:1000, Wako; CD68: 1:200, Abcam). Neurodegeneration was indicated by neuronal loss, neurite degeneration and apoptosis of neurons. Neuronal loss and neurite degeneration in the CA1 region of the hippocampus were detected by double immunofluorescence staining for NeuN and microtubule‐associated protein 2 (Map2) (NeuN: 1:500, Abcam; Map2: 1:2000, Abcam). Apoptosis of neurons in the CA3 region of the hippocampus was assessed by double immunofluorescence staining for NeuN and caspase‐3 (NeuN: 1:500, Abcam; Caspase3: 1:500, Abcam).

Histological staining was performed as described in our previous study (Jin et al., 2017; Wang et al., 2018). All stained sections were photographed with a Zeiss microscope, and staining was photographed and analysed with ImageJ software under the same conditions in a blinded manner to the group information of the sections.

### 4.8 Western blotting

Brain samples were homogenized in ice‐cold RIPA lysis buffer. Protein samples were loaded on a 4%–20% SDS‐polyacrylamide gel and then transferred onto nitrocellulose (NC) membranes. After blocking with 5% fat‐free milk, the NC membranes were incubated with the following primary antibodies overnight at 4°C: anti‐APP C‐terminal (1:1000, Biolegend); anti‐6E10 (1:400, Biolegend); anti‐RAGE (1:1000, Millipore); anti‐LRP‐1 (1:1000, Abcam); anti‐pS396‐tau (1:1000, Abcam); anti‐pT231‐tau (1:1000, Signalway); anti‐Tau5 (1:1000, Millipore); anti‐Synapsin‐1 (1:1000, Abcam); anti‐PSD95 (1:1000, Millipore); and anti‐β‐actin (1:2000, Origene). The membranes were incubated with the corresponding IRDye 800 CW‐conjugated secondary antibodies and scanned using an Odyssey fluorescent scanner. Relative band intensities were normalized to the band intensity of the internal reference protein for analysis.

### 4.9 ELISA

Proteins were extracted from brain tissues in Tris buffer solution (TBS), 2% sodium dodecyl sulfonate (SDS) and 70% formic acid (FA) to measure soluble and insoluble Aβ levels. The levels of human Aβ42 and Aβ40 in the TBS, SDS and FA fractions were measured using ELISA kits (Invitrogen). The levels of inflammatory factors in brain homogenates, including TNF‐α, IL‐1β and IL‐6, were measured with ELISA kits (Raybiotech, USA).

### 4.10 Flow cytometry

Blood was drawn 5 months after spleen excision and anticoagulated with heparin. Flow cytometry analysis of blood immune cells, including T cells, B cells, NK cells, DC cells and monocytes/macrophages, was performed as previously described (Danenberg et al., 2003). In short, red blood cells were lysed (FACS lysing solution, Biolegend) for 9 min at room temperature, and other cells were stained with a BV786‐CD3 antibody, a PE‐CD19 antibody, a PE‐CY7‐NK1.1 antibody, a FITC‐1A/1E antibody, an APC‐CD11b antibody, a BV421‐CD11c antibody, AF‐700‐GR‐1 and 7‐AAD. The cell suspensions were washed twice with PBS containing 1% foetal bovine serum (FBS). All cells were analysed by flow cytometry.

To test the uptake of Aβ, splenic cells were incubated with CD11b microbeads and passed through a magnetic‐activated cell sorting column. CD11b^+^ cells were resuspended in RPMI medium with 10% FBS at a concentration of 10^6^ cells/ml. FITC‐labelled Aβ was added to the cell culture at a concentration of 0.2 µg/ml for 1 hour of incubation at 37°C in a 5% CO2 incubator. Adherent and suspended cells were collected and incubated with TruStainFcX™ PLUS (Biolegend) for Fc receptor blocking. Then, the cells were stained with allophycocyanin (APC)‐conjugated anti‐mouse CD115. Following incubation, the cells were washed with PBS and subjected to flow cytometry (Beckman, USA) after appropriate compensation. The uptake of Aβ was determined by assessing the number of cells that were positive for both FITC and APC.

### 4.11 Statistical analysis

All data are presented as the means ±SEMs. All analyses were conducted with SPSS 22.0 software (SPSS, Chicago, IL, USA). Two‐tailed independent t test, paired t test and the Mann–Whitney U test were used for the comparison of two groups. One‐way ANOVA following the LSD test was used for the multiple comparison in the blood immune cells, Y maze test, annulus crossing and time in target quadrant of MWM test as well as NOR test. Two‐way ANOVA following the LSD test was used to compare two groups at multiple time points for the escape latency of MWM test. *p* < 0.05 was considered statistically significant.

## CONFLICT OF INTEREST

The authors declare that they have no competing interests.

## AUTHOR CONTRIBUTIONS

Y.J.W., X.L.B. and J.W. conceived and designed the project. Z.Y.Y., D.W.C., C.Y.H., G.H.Z. and C.R.T conducted animal and in vitro experiments. Z.Y.Y., D.W.C. and C.R.T analysed data. Z.Y.Y. and Y.J.W. wrote the manuscript.

## Data Availability

The data that support the findings of this study are available from the corresponding author upon reasonable request.
